# Model-based influences on humans’ choices and striatal prediction errors

**DOI:** 10.1016/j.neuron.2011.02.027

**Published:** 2011-03-24

**Authors:** Nathaniel D. Daw, Samuel J. Gershman, Ben Seymour, Peter Dayan, Raymond J. Dolan

**Affiliations:** 1 Center for Neural Science and Department of Psychology, New York University; 2 Department of Psychology and Neuroscience Institute, Princeton University; 3 Wellcome Trust Centre for Neuroimaging, Institute of Neurology, University College London; 4 Gatsby Computational Neuroscience Unit, University College London

## Abstract

The mesostriatal dopamine system is prominently implicated in model-free reinforcement learning, with fMRI BOLD signals in ventral striatum notably covarying with model-free prediction errors. However, latent learning and devaluation studies show that behavior also shows hallmarks of model-based planning, and the interaction between model-based and model-free values, prediction errors and preferences is underexplored. We designed a multistep decision task in which model-based and model-free influences on human choice behavior could be distinguished. By showing that choices reflected both influences we could then test the purity of the ventral striatal BOLD signal as a model-free report. Contrary to expectations, the signal reflected both model-free and model-based predictions in proportions matching those that best explained choice behavior. These results challenge the notion of a separate model-free learner and suggest a more integrated computational architecture for high-level human decision-making.

## Introduction

An ubiquitous idea in psychology, neuroscience, and behavioral economics is that the brain contains multiple, distinct systems for decision-making (Daw et al., 2005; Kahneman, 2003; Loewenstein and O’Donoghue, 2004; Rangel et al., 2008; Redish et al., 2008; Sloman, 1996). One prominent contender dates back to Thorndike’s (1911) “law of effect,” which states that an action followed by reinforcement is more likely to be repeated in the future. This habit principle is also at the heart of temporal-difference (TD) learning accounts of the dopaminergic system and its action in striatum (Barto, 1995; Schultz et al., 1997). In the actor-critic, for instance, a dopaminergic “reward prediction error” (RPE) signal plays the role of Thorndike’s reinforcer, increasing the propensity to take actions that are followed by positive RPEs (Maia, 2010; Suri and Schultz, 1999).

However, it has long been known that the reinforcement principle offers at best an incomplete account of learned action choice. Evidence from reward devaluation studies suggests that animals can also make “goal-directed” choices, putatively controlled by representations of the likely outcomes of their actions (Dickinson and Balleine, 2002). This realizes a suggestion, dating back at least to Tolman (1948), that animals are not condemned merely to repeat previously reinforced actions.

From the perspective of neuroscience, habits and goal-directed action systems appear to coexist in different corticostriatal circuits. While these systems learn concurrently, they control behavior differentially under alternative circumstances (Balleine and O’Doherty, 2010; Dickinson, 1985; Killcross and Coutureau, 2003). Computational treatments (Balleine et al., 2008; Daw et al., 2005; Doya, 1999; Niv et al., 2006; Redish et al., 2008) interpret these as two complementary mechanisms for reinforcement learning (RL). The TD mechanism is associated with dopamine and RPEs, and is “model-free” in the sense of eschewing the representation of task structure and instead working directly by reinforcing successful actions. The goal-directed mechanism is a separate “model-based” RL system, which works by using a learned “internal model” of the task to evaluate candidate actions (e.g., by mental simulation; Hassabis and Maguire, 2007; Schacter et al., 2007; perhaps implemented by some form of preplay; Foster and Wilson, 2006; Johnson and Redish, 2007).

Bar one recent exception (Gläscher et al., 2010) (which focused on the different issue of the neural substrates of learning the internal model), previous studies investigating the neural substrates of model-free and model-based control have not attempted to detect simultaneous correlates of both as these systems learn concurrently. Thus, the way the controllers interact is unclear, and the prevailing supposition that neural RPEs originate from a distinct model-free system remains untested. Here we exploited the difference between their two types of action evaluation to investigate the interaction of the controllers in humans quantitatively, using functional MRI. Model-free evaluation is retrospective, chaining RPEs backward across a sequence of actions. By contrast, model-based evaluation is prospective, directly assessing available future possibilities. Thus, it is possible to distinguish the two using a sequential choice task.

In theory, the choices recommended by model based and model free strategies depend on their own, separate, valuation computations. Thus, if behavior reflects contributions from each strategy, then we can make the clear, testable, prediction that neural signals reflecting either valuation should dissociate from behavior (Kable and Glimcher, 2007). Correlates of reward prediction have most repeatedly been demonstrated in fMRI in two areas: the ventromedial prefrontal cortex (vmPFC) and the ventral striatum (ventral putamen and nucleus accumbens) (Delgado et al., 2000; Hare et al., 2008; Knutson et al., 2007; Knutson et al., 2000; Lohrenz et al., 2007; O’Doherty, 2004; Peters and Buchel, 2009; Plassmann et al., 2007; Preuschoff et al., 2006; Tanaka et al., 2004; Tom et al., 2007). Of these, value-related signals in medial prefrontal cortex are sensitive to task contingencies, and are thus good candidates for involvement in model-based evaluation (Hampton et al., 2006, 2008; Valentin et al., 2007). Conversely, the ventral striatal signal correlates with an RPE (McClure et al., 2003a; O’Doherty et al., 2003; Seymour et al., 2004), and on standard accounts, is presumed to be associated with dopamine and with a model-free TD system. If so, these signals should reflect *ignorance* of task structure and instead be driven by past reinforcement, even though subjects’ behavior, if it is partly under the control of a separate model-based system, may be better informed.

Contrary to this hitherto untested prediction, our results demonstrate that reinforcement-based and model-based value predictions are combined in both brain areas, and more particularly, that RPEs in ventral striatum do not reflect pure model-free TD. These results suggest a more integrated computational account of the neural substrates of valuation.

## Results

### Behavior

**Figure 1:**
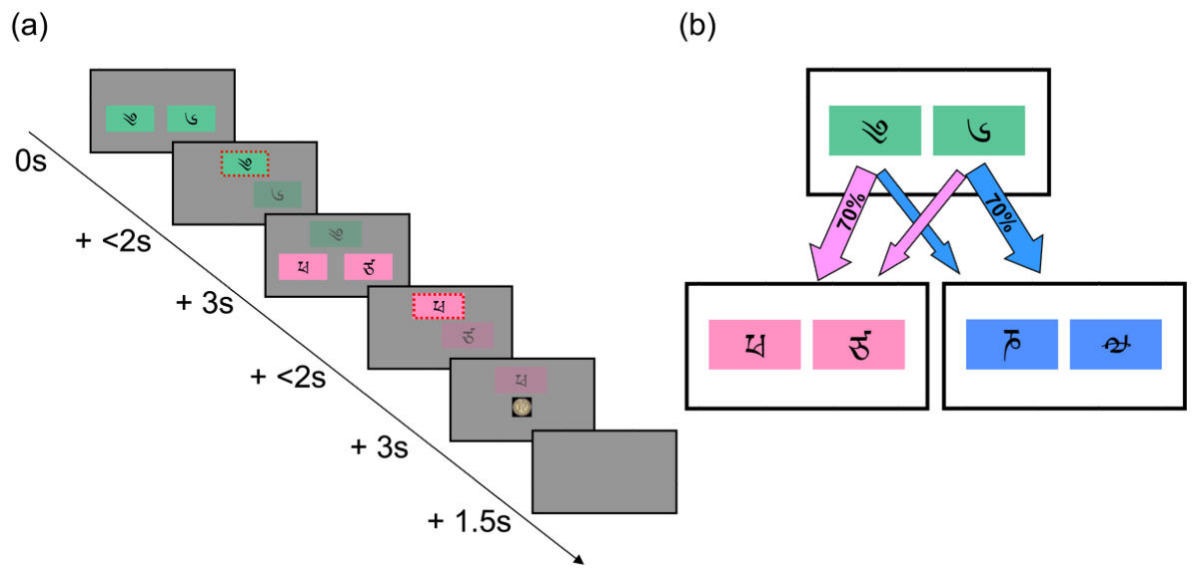
(a) Timeline of events in trial. A first-stage choice between two options (green boxes) leads to a second-stage choice (here, between two pink options), which is reinforced with money. (b) State transition structure. Each first-stage choice is predominantly associated with one or the other of the second-stage states, and leads there 70% of the time.

Subjects (n=17) completed a two-stage Markov decision task (Figure 1) in which, on each trial, an initial choice between two options labeled by (semantically irrelevant) Tibetan characters led probabilistically to either of two, second-stage “states,” represented by different colors. In turn, these both demanded another two-option choice, each of which was associated with a different chance of delivering a monetary reward. The choice of one first-stage option led predominantly (70% of the time) to an associated one of the two second-stage states, and this relationship was fixed throughout the experiment. However, to incentivize subjects to continue learning throughout the task, the chances of payoff associated with the four second-stage options were changed slowly and independently, according to Gaussian random walks. Theory (Daw et al., 2005; Dickinson, 1985) predicts that such change should tend to favor the ongoing contribution of model-based evaluation.

Each subject undertook 201 trials, of which 2 ± 2 (mean ± 1 SD) trials were not completed due to failure to enter a response within the two second limit. These trials were omitted from analysis.

The logic of the task was that model-based and model-free strategies for RL predict different patterns by which reward obtained in the second stage should impact first-stage choices on subsequent trials. For illustration, consider a trial in which a first-stage choice, uncharacteristically, led to the second-stage state with which it is not usually associated, and in which the choice then made at the second stage was rewarded. The principle of reinforcement would predict that this experience should increase the probability of repeating the first-stage choice since it was ultimately rewarded. However, a subject choosing instead using an internal model of the task’s transition structure, that evaluates actions prospectively, would be expected instead to *decrease* the probability of choosing that same option. This is because any increase in the value of the rewarded second-stage option will more greatly increase the expected value of the first-stage option that is more likely to lead there. This is actually the first-stage option that was not originally chosen.

**Figure 2:**
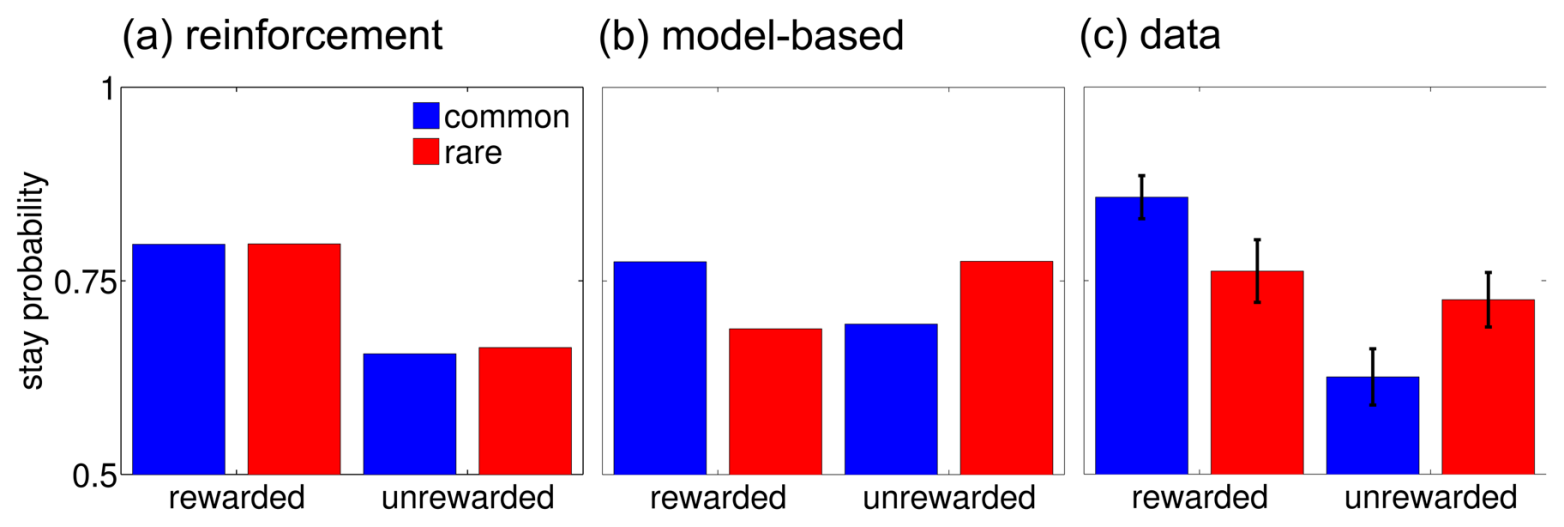
Factorial analysis of choice behavior. (a) Simple reinforcement predicts that a first-stage choice resulting in reward is more likely to be repeated on the subsequent trial, regardless of whether that reward occurred after a common or rare transition. (b) Model-based prospective evaluation instead predicts that a rare transition should affect the value of the other first-stage option, leading to a predicted interaction between the factors of reward and transition probability. (c) Actual stay proportions, averaged across subjects, display hallmarks of both strategies. Error bars: 1 SEM.

Given previous work suggesting the coexistence of multiple valuation processes in the brain (Balleine et al., 2008; Dickinson, 1985), we hypothesized that subjects might exhibit a mixture of both strategies. First, to see learning effects of this sort in a relatively theory-neutral manner, we directly assessed the effect of events on the previous trial (trial *n*) on the choice on the current trial (trial *n+1*). The two key events on trial *n* are whether or not reward was received, and whether the second-stage state presented was the common or rare, given the first-stage choice on trial *n*. We evaluated the impact of these events on the chance of repeating the same first-stage choice on trial *n*+*1*. For reasons outlined above, a simple reinforcement strategy (simulated in Figure 2a using the TD algorithm SARSA(λ) for λ=1) predicts only a main effect of reward: an ultimately rewarded choice is more likely to be repeated, regardless of whether that reward followed a common or rare transition. Conversely, a model-based strategy (simulated in Figure 2b) predicts a crossover interaction between the two factors, because a rare transition inverts the effect of the subsequent reward.

Figure 2c plots the observed choice proportions as a function of these two factors, in the average across subjects. In order to study effects that were statistically reliable at the level of the population, we quantified the effects using hierarchical logistic regression with all coefficients taken as random effects across subjects. At the population level, the main effect of reward was significantly different from zero (p<1e-8, two-tailed), demonstrating a reinforcement effect. However, the interaction between reward and the transition probability was also significant (p<5e-5), rejecting a pure reinforcement account and suggesting that subjects take the transition model into account in making their choices. As both theories predict, there was no significant main effect of transition likelihood (p=.5). Finally, the constant term was significantly positive (p<5e-12), suggesting an overall tendency to stick with the same option from trial to trial, notwithstanding reward (Ito and Doya, 2009; Kim et al., 2009; Lau and Glimcher, 2005). We also considered estimates of the effect sizes for each individual within this analysis (conditional on the group level parameter estimates); the effect of reward was positive (within the 95% confidence interval) for 14/17 subjects, and the interaction was positive for 10/17 individuals, including 7 for whom the main effect of reward was also positive. Together these data suggest that hallmarks of both strategies are seen significantly at the population level and within many individuals, but that there may be between-subject variability in their deployment.

Motivated by these results, we considered the fit of full model-based and model-free (SARSA(λ) TD; Rummery and Niranjan, 1994) RL algorithms to the choice sequences. The former evaluates actions by prospective simulation in a learned model; the latter uses a generalized principle of reinforcement. The generalization, controlled by the reinforcement eligibility parameter λ, is that the estimated value of the second-stage state should act as the same sort of model-free reinforcer for the first-stage choice as the final reward actually received after the second-stage choice. The parameter λ governs the relative importance of these two reinforcers, with λ=1 being the special case of figure 2a in which only the final reward is important, and λ=0 being the purest case of the TD algorithm in which only the second-stage value plays a role.

We also considered a hybrid theory (Gläscher et al., 2010) in which subjects could run both algorithms in parallel, and make choices according to the weighted combination of the action values that they produce (see *Experimental Procedures*). We took the relative weight of the two algorithms’ values in determining the choices to be a free parameter, which we allowed to vary across subjects but assumed to be constant throughout the experiment. Thus, this algorithm contains both the model-based and TD algorithms as special cases, where one or the other gets all weight. We first verified that the model fit significantly better than chance; it did so, at p<.05, for all 17 subjects (likelihood ratio tests).

We estimated the theory’s free parameters individually for each subject by maximum likelihood (Table 1). Such an analysis treats each subject as occupying a point on a continuum trading off the two strategies; tests of the parameter estimates across subjects seek effects that are generalizable to other members of the population (analogous to the random effects level in fMRI; Holmes and Friston, 1998). Due to non-Gaussian statistics (since the parameters are expected to lie in the unit range), we analyzed the estimated parameters’ medians using nonparametric tests. Across subjects, the median weighting for model-free RL values was 61% (with model-based RL at 39%), which was significantly different from both 0 and 100% (sign tests, Ps<.005), again suggesting both strategies were mixed in the population. The second important parameter is the reinforcement eligibility parameter λ, which controls the two reinforcement effects in TD, i.e., the relative influence of the estimated value of the second-stage state and the ultimate reward on the model-free value of the first-stage choice. Across subjects, the median estimate for λ was 0.57 (significantly different from 0 and 1; sign tests, Ps<.05), suggesting that at the population level reinforcement occurred in part according to TD-like value chaining (λ<1) and in part according to direct reinforcement (λ>0).

Since analyzing estimates of the free parameters does not speak to their necessity for explaining data, we used both classical and Bayesian model comparison to test whether these free parameters of the full model were justified by data, relative to four simplifications. We tested the special cases of TD(λ) and model-based RL alone, plus the hybrid model using only direct reinforcement or value chaining (i.e., with λ restricted to 0 or 1). The results in Table 2 show the superiority of the hybrid model both in the aggregate over subjects and also, in most tests, for the majority of subjects considered individually. Finally, we fit the hierarchical model of Stephan et al. (2009) to treat the identity of the best fitting model as a random effect that itself could vary across subjects. The exceedance probabilities from this analysis, shown in Table 2, indicate that the hybrid model had the highest chance (with probability 92%) of being the most common model in the population. The same analysis estimated the expected proportion of each sort of learner in the population; here the hybrid model was dominant (at 48%), followed by TD at 18%.

Together, these analyses provided compelling support for the proposition that the task exercised both model-free and model-based learning strategies, albeit with evidence for individual variability in the degree to which subjects deploy each of them. Next, armed with the trial-by-trial estimates of the values learned by each putative process, from the hybrid algorithm (refit using a mixed-effects model for more stable fMRI estimates; Table 3), we sought neural signals related to these valuation processes.

### Neuroimaging

BOLD responses in a number of regions – notably the striatum and the ventromedial prefrontal cortex (vmPFC) – have repeatedly been shown to covary with subjects’ value expectations (Berns et al., 2001; Hare et al., 2008; O’Doherty et al., 2007). The ventral striatum has been closely associated with model-free RL, and so a prime question is whether BOLD signals in this structure indeed reflect model-free knowledge alone, even for subjects whose actual behavior shows model-based influences.

To investigate this question, we sought voxels where BOLD activity correlated with two candidate timeseries. The first timeseries was the standard RPE based on model-free TD, using just the timepoints of the transition to the second-stage and the delivery of the outcome in order to avoid uncertainty about the appropriate baseline against which to measure the first-stage prediction (see Supplemental Experimental Procedures). The second timeseries involved subtracting these TD prediction errors from the RPEs that would arise if the predictions had been model-based rather than model free (Daw, in press; Friston et al., 1998; Wittmann et al., 2008).

We adopted this approach (rather than simply including both model-free and model-based RPEs as explanatory variables) to reduce the correlation between the regressors of interest, and also because it encompassed the test of the null hypothesis that RPE signaling in striatum was purely model-free. If so, then the signal would be accounted for entirely by the model-free regressor, and the difference timeseries should not correlate significantly. If, however, the BOLD signal reflected pure model-based values, or any combination of both, then it would be best described by some weighted combination of the two regressors; that is, the difference regressor would account for residual BOLD activity in addition to that accounted for by the model-free RPE. We tested the conjunction of the two regressors to verify whether BOLD activity in a voxel was indeed significantly correlated with the weighted sum of both (Nichols et al., 2005).

**Figure 3:**
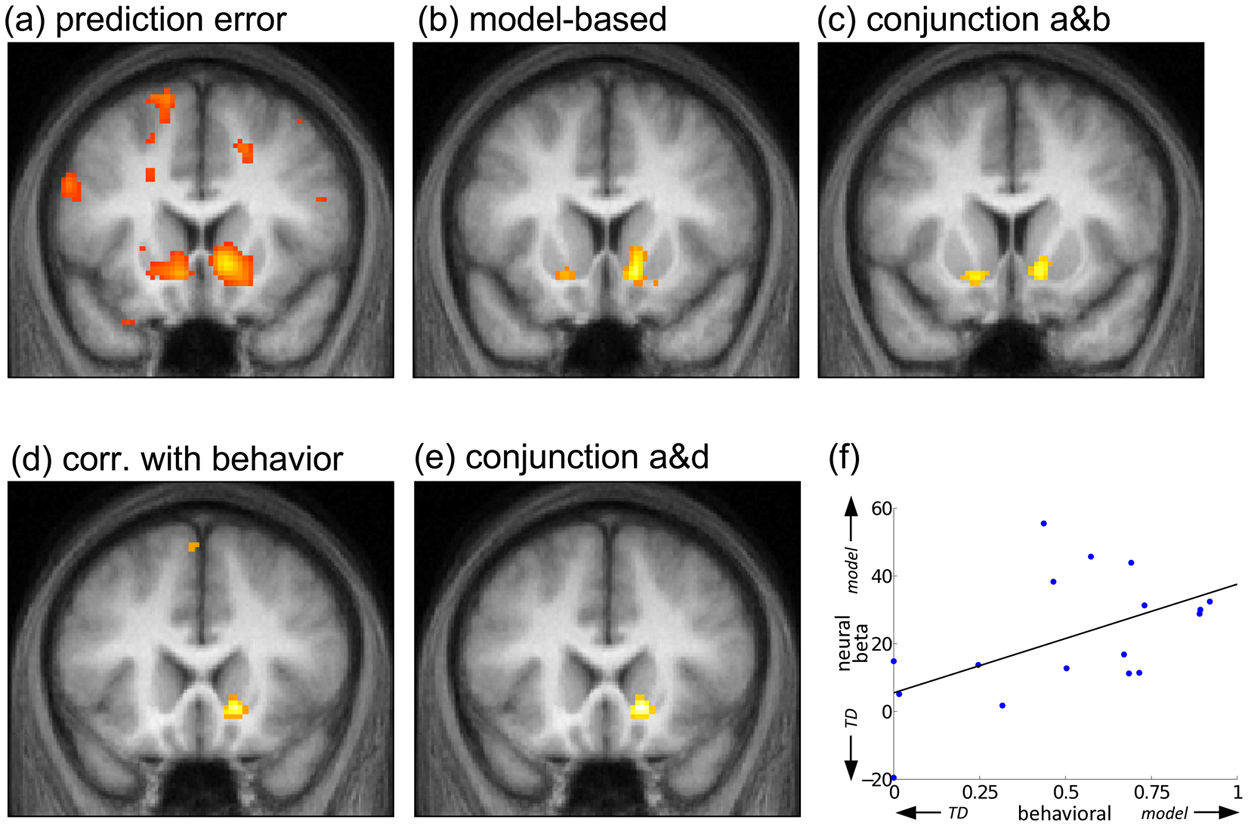
Neural correlates of model-free and model-based valuations in RPE in striatum. All maps thresholded at p<.001 uncorrected for display. (a) Correlates of model-free RPE in bilateral striatum (left peak: −12 10 4, right: 10 12 −4). (b) RPE signaling in ventral striatum is better explained by including some model-based predictions: correlations with the difference between model-based and model-free RPE signals (left: −10 6 12, right: 12 16 −8). (c) Conjunction of contrasts from a and b (left: −12 10 −10, right, 12 16 −6). (d) Region of right ventral striatum where the weight given to model-based valuations in explaining the BOLD response correlated, across subjects, with that derived from explaining their choice behavior (14 20 −6). (e) Conjunction of contrasts from a and d (14 20 −6). (f) Scatterplot of the correlation from d, from average activity over an anatomically defined mask of right ventral striatum. (r^2^ =.28, p=.027).

**Figure 4:**
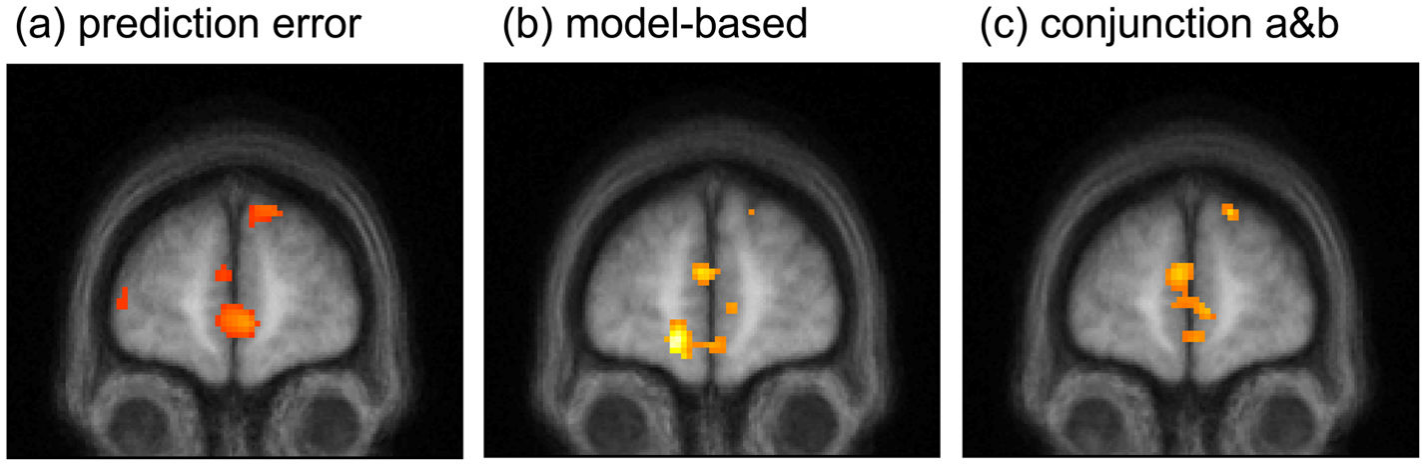
Neural correlates of model-free and model-based valuations in RPE in medial PFC. Thresholded at p<.001 uncorrected (a and b) or p<.005 uncorrected (c) for display. (a) Correlates of model-free RPE in medial PFC (−4 66 14). (b) RPE signaling in medial PFC is better explained by including some model-based predictions: correlations with the difference between the two RPE signals (−4 56,14). (c) Conjunction of contrasts from a and b (−4 62 12).

Figure 3a shows that BOLD activity correlated significantly with the model-free RPE timeseries in left and right ventral striatum (both p<.001; except where noted, all reported statistics are corrected at the cluster level for familywise error due to whole-brain multiple comparisons). Moreover, this activity was better characterized, on average, as including some model-based valuation: the model-based difference regressor loaded significantly (right, p<.005, left, p<.05; Figure 3b) in the same area (conjunction; right, p<.01 whole-brain corrected; left, p<.01 small-volume corrected within an anatomically defined mask of the bilateral nucleus accumbens; Figure 3c). Similar results, though less strong, were also observed in medial/ventromedial prefrontal cortex (vmPFC), where both model-free RPE (p<.001; Figure 4a) and the difference regressor indicating model-based valuation (p<.01; Figure 4b) correlated significantly with BOLD activity. However, although the conjunction between these two maps showed voxels significant at p<.001 uncorrected, it survived whole-brain multiple comparison correction for cluster size (at p<.005 corrected; Figure 4c) only when the threshold on the conjunction map was relaxed to p<.005 uncorrected. (Note that cluster size correction is valid independent of the threshold on the underlying uncorrected map, though examining additional thresholds implies additional multiple comparisons; Friston et al., 1993.)

These results suggested that RPE-related BOLD signals in ventral striatum, and also vmPFC, reflected valuations computed at least in part by model-based methods rather than pure TD. To investigate this activity further, we compared neural and behavioral estimates of the degree of reliance on model-based valuation, across subjects. The neural and behavioral estimates should correlate if, though computed using different observables, they were measuring the same phenomenon, and if RPE activity in striatum was related to a behaviorally relevant mixture of model-based and model-free values, rather than to one or the other. We measured the degree of model-based valuation in the neural signal by the effect size estimated for the model-based difference regressor (with a larger weighting indicating that the net signal represented an RPE more heavily weighted toward model-based values). Behaviorally, we assessed the degree of model-based influence on choices by the fit of the weighting parameter *w* in the hybrid algorithm. Significant correlation between these two estimates was indeed detected in right ventral striatum (p<.01 small volume corrected within an anatomical mask of bilateral nucleus accumbens; Figure 3d); and the site of this correlation overlapped the basic RPE signal there (p<.01 small volume corrected; Figure 3e). Figure 3f illustrates a scatterplot of the effect, here independently re-estimated from BOLD activity averaged over an anatomically defined mask of right nucleus accumbens. The finding of consistency between both these estimates helps to rule out unanticipated confounds specific to either analysis.

Altogether, these results suggested that BOLD activity in striatum reflected a mixture of model-free and model-based evaluations, in proportions matching those that determine choice behavior. Finally, in order more directly to characterize this activity and to interrogate this conclusion via an analysis using different data-points and weaker theoretical assumptions, we subjected BOLD activity in ventral striatum to a factorial analysis of its dependence on the previous trial’s events, analogous to that used for choice behavior in Figure 2. In particular, the TD RPE when a trial starts reflects the value expected during the trial (as in the anticipatory activity of Schultz et al., 1997), which can be quantified as the predicted value of the top-level action chosen (Morris et al., 2006). For reasons analogous to those discussed above for choice behavior, learning by reinforcement as in TD(λ) (for λ>0) predicts that this value should reflect the reward received following the same action on the previous trial. However, a model-based valuation strategy instead predicts that this previous reward effect should interact with whether the previous choice was followed by a common or rare transition.

**Figure 5:**
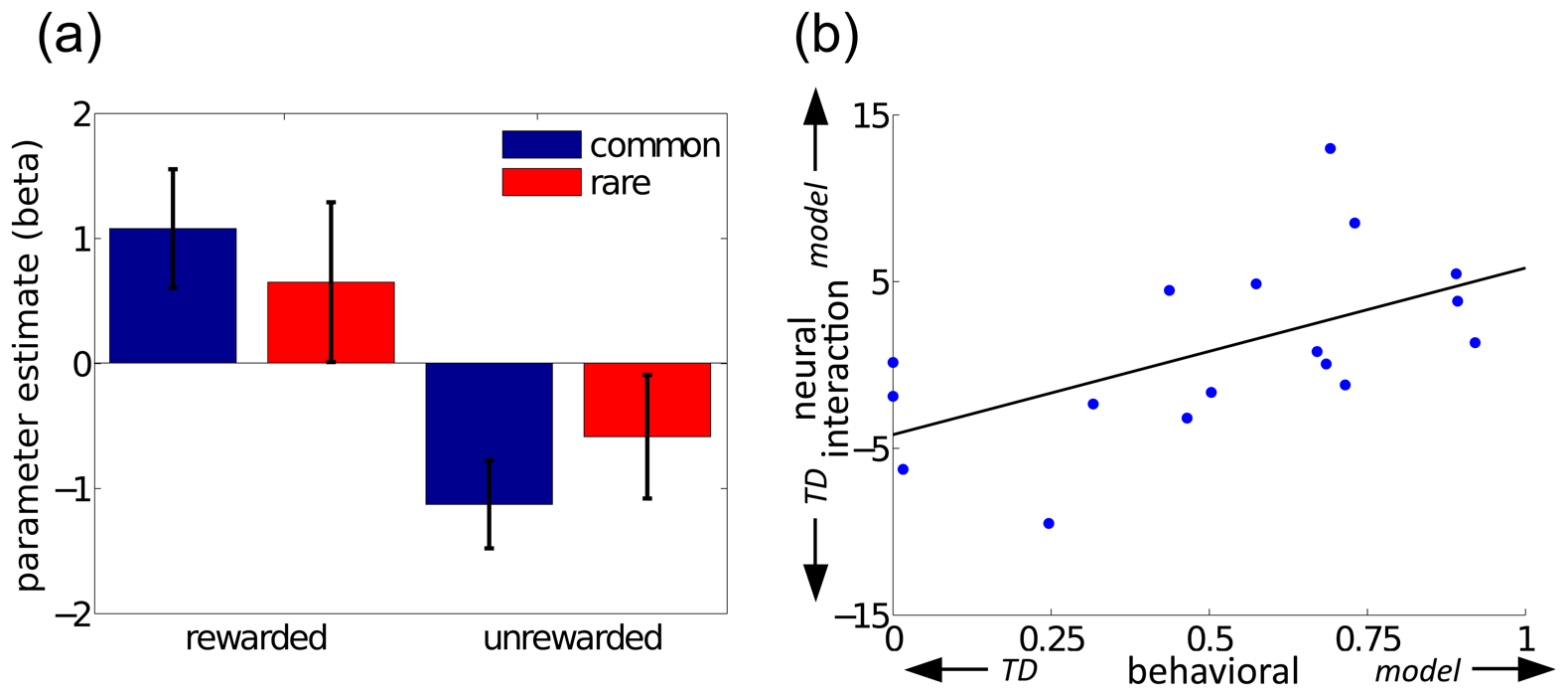
Factorial analysis of BOLD signal at start of trial, from average activity over an anatomical mask of right nucleus accumbens. (a) Signal change (relative to mean) as a function of whether the choice on the previous trial previous trial was rewarded or unrewarded, and whether that occurred after a common or rare transition (compare Figure 2c) Error bars: 1SEM. (b) Scatterplot of the correlation, across subjects, between the contrast measuring the size of the interaction between reward and transition probability (an index of model-based valuation), and the weight given to model-based vs model-free valuations in explaining choice behavior. (r^2^=0.32, p=.017).

We therefore examined BOLD activity at the start of trials in right ventral striatum (defined anatomically), as a function of the reward and transition on the previous trial. For reasons mentioned above, these signals did not form part of the previously described parametric RPE analyses. In order to isolate activity specifically related to the same action that had been learned about on the previous trial, we restricted our assessment to those trials in which the same action was chosen twice in a row (Morris et al., 2006). As seen in Figure 5a, there was a main effect of reward (p<.005), consistent with TD-like valuation. This, to our knowledge, is the first time that RPEs in BOLD have been directly shown to exhibit learning through an explicit dependence on previous-trial outcomes (Bayer and Glimcher, 2005). Across subjects, the interaction with the transition probability – the marker for model-based evaluation – was not significant (p>.4), but the size of the interaction per-subject (taken as another neural index of the per-subject model-based effect) correlated with the behavioral index of model-based valuation (p<.02; Figure 5b). This last result further confirmed that striatal BOLD reflected model-based valuation to the extent that choice behavior did. Indeed, speaking to the consistency of the results, although the two neural estimates reported here for the extent of model-based valuation in the striatal BOLD signal (Figures 3f and 5b) were generated from different analytical approaches, and based on activity modeled at different timepoints within each trial, they significantly correlated with one another (r^2^=0.37; p<.01).

## Discussion

We studied human choice behavior and BOLD activity in a two-stage decision task that allowed us to disambiguate model-based and model-free valuation strategies through their different claims about the effect of second-stage reinforcement on first-stage choices and BOLD signals. Here, ongoing adjustments in the values of second-stage actions extended the one-shot reward devaluation challenge often used in animal conditioning studies (Dickinson, 1985) and also the introduction of novel goals as in latent learning (Gläscher et al., 2010): they continually tested whether subjects prospectively adjusted their preferences for actions leading to a subsequent incentive (here, the second-stage state), when its value changed. Following Daw et al., (2005), we see such reasoning via sequential task structure as the defining feature that distinguishes model-based from model-free approaches to RL (although Hampton et al., 2006, and Bromberg-Martin et al., 2010 hold a somewhat different view: they associate model-based computation with learning nonsequential task structure as well).

We recently used a similar task in a complementary study (Gläscher et al., 2010) which minimized learning about the rewards (by reporting them explicitly and keeping them stable), to isolate learning about the state transition contingencies. Here by contrast, we minimized transition learning (by partly instructing subjects) and introduced dynamic rewards to allow us to study the learning rules by which neural signals tracked them. This, in turn, allowed us to test an uninvestigated assumption of the analysis in the previous paper, i.e. the isolation of model-free value learning as expressed in the striatal PE.

Our previous computational theory of multiple RL systems in the brain (Daw et al., 2005) focused on a dynamic mechanism for trading off the reliance on model-based and model-free valuations based on their relative uncertainties. In the current task, the ever-changing rewards should keep the trade-off roughly constant over time, allowing us to focus on the broader two-system structure of this theory. Rather than confronting the many (unknown) factors that determine the uncertainties of each system within each subject, we treated the balance between the two processes as exogenous, controlled by a constant free parameter (*w*), whose value we could estimate. Indeed, consistent with our intent, there was no significant trend (analyses not presented) towards progressive habit formation (Adams, 1982; Gläscher et al., 2010).

Nevertheless, consistent with findings from animal learning (Balleine and O’Doherty, 2010; Balleine et al., 2008; Dickinson, 1985; Dickinson and Balleine, 2002), we found clear evidence for both TD- and model-like valuations, suggesting that the brain employs a combination of both strategies. The standard view is that the two putative systems work separately and in parallel, a view reinforced by the strong association of the mesostriatal dopamine system with model-free RL and the fact that, in animal studies, each system appears to operate relatively independently when brain areas associated with the other are lesioned (Killcross and Coutureau, 2003; Yin et al., 2004; Yin et al., 2005). Also consistent with this idea, previous work (Hampton et al., 2006, 2008) suggested model-based influences on the vmPFC expected value signal, but did not test for additional model-free influences there, nor conversely whether model-based influences also affected striatal RPEs. Here we found that even the signal most associated with model-free RL, the striatal RPE, reflects both types of valuation, combined in a way that matches their observed contribution to choice behavior. The finding that a similar result in vmPFC was weaker may reflect the fact that neural signaling there is, in some studies, better explained by a correlated variable, expected future value, and not RPE per se (Hare et al., 2008); residual error due to such a discrepancy could suppress effects there. However, in a sequential task these two quantities are closely related, thus, unlike Hare’s, the present study was not designed to dissociate them.

Our ventral striatal finding invites a re-evaluation of the standard account of RPE signaling in the brain, since it suggests that even a putative TD system does not exist in isolation from model-based valuation. One possibility about what might replace this account is suggested by contemplating an infelicity of the algorithm used here for data analysis. In order to reject the null hypothesis of purely model-free RPE signaling, we defined a generalized RPE with respect to model-based predictions as well. However, this augmented signal was nugatory, in the sense that model-based RPEs played no role in our account of choice behavior. Indeed, model-based learners do not rely on model-based RPEs: the learning problem they face – tracking state transition probabilities and immediate rewards rather than cumulative future rewards – demands different training signals (Gläscher et al., 2010).

This apparent mismatch encourages consideration of a hybrid of a different sort. We have so far examined theories in which model-based and model-free predictions compete directly to select actions (Daw et al., 2005). However, model-based and model-free RPEs could also usefully be integrated for training. For instance, consider the standard actor-critic account (Barto et al., 1983; Barto, 1995). This uses RPEs derived from model-free predictions (the critic) to reinforce action selection policies (the actor). Errors in model-based predictions, if available, could serve the same purpose. A model-free actor trained, in part, by such a “model-based” critic would, in effect, cache (Daw et al., 2005) or memorize the recommendations of a model-based planner, and could execute them subsequently without additional planning.

The computational literature on RL includes some related ideas, in algorithms such as prioritized sweeping (Moore and Atkeson, 1993), which caches the results of model-based evaluation (albeit without a model-free component), and Dyna (Johnson and Redish, 2005; Sutton, 1990) which trains a model-free algorithm (though offline) using simulated experiences generated from a world model. In neuroscience, various theories have been proposed in which a world model impacts the input to the model-free system (Bertin et al., 2007; Daw et al., 2006a; Doya, 1999; Doya et al., 2002). The architecture suggested here more closely resembles the “biased” learning hypothesized by Doll et al. (2009), according to which top-down information (there provided by experimenter instructions rather than a learned world model) modifies the target of model-free RL. Outside the domain of learning, striatal BOLD responses are indeed affected by values communicated by instruction rather than experience (Fitzgerald et al., 2010; Tom et al., 2007) and also by emotional self-regulation (Delgado et al., 2008).

Further theoretical work is needed to characterize the different algorithms suggested by this general architecture. However, in general, by preserving the overall structure of parallel model-based and model-free systems – albeit exchanging information at an earlier level – the proposal of a model-based critic would appear to remain consistent with the lesion data suggesting that the systems can function in isolation (Killcross and Coutureau, 2003; Yin et al., 2004; Yin et al., 2005) and with behavioral data demonstrating that distinct decision systems may have different properties and can be differentially engaged in different circumstances (Doeller and Burgess, 2008; Frank et al., 2007; Fu and Anderson, 2008). It also remains consistent with other fMRI studies (Doeller et al., 2008; Poldrack et al., 2001; Venkatraman et al., 2009) suggesting that overall activity in different brain systems associated with either system can modulate with time or circumstances, presumably in relation to the extent that either process is engaged.

Apart from training, a different use for model-based RPEs would be for online action evaluation and selection. In particular, Doya (1999) proposed that a world model could be used to predict the next state following a candidate action, and that a dopaminergic RPE with respect to that projected state could then be used to evaluate whether the action was worth taking (in a scheme related to that suggested by McClure et al., 2003b; Montague et al., 1995; Montague et al., 1996). RPEs for planning would appear to be categorically different in timing and content than RPEs for learning, in that the former are triggered by hypothetical state transitions and the latter by actual ones, as in the effects reported here. The Doya (1999) circuit also differs from a full model-based planner in that it envisions only a single step of model-based state lookahead; however, to test this limitation would require a task with longer sequences.

In the present study, as in most fMRI studies of RPEs, our effects focused on ventral striatum, and we did not see any correlates of the organization of striatum into components associated with different learning strategies as suggested by the rodent literature (Yin et al., 2004; Yin et al., 2005). Further, although there is evidence suggesting that RPE effects in the ventral striatal BOLD signal reflect, at least in part, dopaminergic action there (Knutson and Gibbs, 2007; Pessiglione et al., 2006; Schonberg et al., 2010), the BOLD signal in striatum likely conflates multiple causes including also cortical input and local activity, and it is thus not possible to identify it uniquely with dopamine. Indeed, it is possible, even if the effects attributed to our model-free RPE regressor are dopaminergic in origin, that the residual effects captured by the model-based difference regressor in the same voxels arise from other sources. The questions raised by the present study thus invite resolution by testing a similar multistep task in animals using dopamine unit electrophysiology or voltammetry. In this respect, recent results by Bromberg-Martin et al. (2010) showing that in a serial reversal task (albeit nonsequential) a dopaminergic RPE response is more sophisticated than a basic TD theory would predict, provides a tantalising clue that our results might hold true of dopaminergic spiking as well.

Overall, by demonstrating that it is feasible to detect neural and behavioral signatures of both learning strategies, the present study opens the door to future within-subject studies targeted at manipulating and tracking the tradeoff dynamically, and thence, at uncovering the computational mechanisms and neural substrates for controlling it. Such meta-control of decision systems is of particular practical importance, for instance because the compulsive nature of drug abuse has been proposed to result from aberrant expression of habitual control (Everitt and Robbins, 2005), and similar mechanisms have also, plausibly, been linked to other serious issues of self-control, including undersaving and overeating (Loewenstein and O’Donoghue, 2004).

## Experimental Procedures

### Participants and behavioral task

Seventeen healthy adults (five female; mean age 25.8 years) participated in this study. All participants gave written informed consent, and the study was conducted in accordance with the guidelines of the local ethics committee.

The task consisted of 201 trials, in three blocks of 67, separated by breaks. The events in the trial are sketched in Figure 1a. Each trial consisted of two stages. In the first stage, subjects used an MR compatible button box to choose between two options, represented by Tibetan characters in colored boxes. If subjects failed to enter a choice within 2 seconds, the trial was aborted. The chosen option rose to the top of the screen, while the option not chosen faded and disappeared. At the second stage, subjects were presented with either of two more choices between two options (“states”), and entered another choice. The second choice was rewarded with money (depicted by a pound coin, though subjects were paid 20% of this amount), or not (depicted by a zero). Trials were separated by an inter-trial interval of randomized length, on average about 1TR.

Which second-stage state was presented depended, probabilistically, on the first-stage choice, according to the transition scheme shown in Figure 1b. The assignment of colors to states was counterbalanced across subjects, and the two options at each state were permuted pseudorandomly between left and right from trial to trial. Each bottom-stage option was rewarded according to a probability associated with that option. In order to encourage ongoing learning, these reward probabilities were diffused at each trial by adding independent Gaussian noise (mean 0, SD .025), with reflecting boundaries at .25 and .75.

In a computerized training session prior to the fMRI task, subjects were instructed that the reward probabilities would change, but those controlling the transitions from the first to the second stage would remain fixed. They were also instructed about the overall structure of the transition matrix, specifically, that each first stage option was primarily associated with one or the other of the second-stage states, but not which one. Prior to the scanning session, to familiarize themselves with the structure of the task, subjects played 50 trials on a practice task using a different stimulus set.

### Behavioral analyses

We first conducted a logistic regression in which the dependent variable was the first stage choice (coded as stay vs switch), and the explanatory variables were the reward received on the previous trial, a binary indicator variable indicating whether the previous trial’s transition was common or rare, and the interaction of the two. We took all coefficients as random effects across subjects, and estimated this multilevel regression using the lme4 linear mixed effects package (Bates and Maechler, 2010) in the R statistical language (R Development Core Team, 2010). We also extracted posterior effect size estimates (conditional on the estimated population-level prior) and confidence intervals from the posterior covariance for each of the individuals from this fit. The predictions in Figures 2a,b are derived from simulations of SARSA(1) and model-based algorithms (below), using the parameters best fit to the subjects’ data within each class of algorithm.

### Computational model of behavior

In a second set of analyses, we fit choice behavior to an algorithm that is similar to the hybrid algorithm of Gläscher et al. (2010). In particular, it learned action values via both model-based RL (explicit computation of Bellman’s equation) and by model-free SARSA(λ) TD learning (Rummery and Niranjan, 1994), and assumed choices were driven by the weighted combination of these two valuations. The relative weighting was controlled by a free parameter *w*, which we assumed to be constant across trials. We also computed TD RPEs with respect to both the model-free and model-based valuations, and, for fMRI analysis, defined a “difference regressor” as the difference between them. Full equations are given in Supplemental Experimental Procedures.

### Behavioral estimation

For behavioral analysis, we estimated the free parameters of the algorithm separately for each subject, to maximize the log-likelihood of the data (from the log of Equation 2 summed over all trials), for the choices actually made conditioned on the states and rewards previously encountered. We constrained the learning rates to lie between zero and one, but allowed λ and *w* (which also nominally range between zero and one) to float arbitrarily beyond these boundaries, so as to make meaningful the tests whether the median estimates were different from the nominal boundaries across the population.

For classical model comparison, we repeated this procedure for the nested subcases, and tested the null hypothesis of the parametric restriction (either individually per subject, or for likelihoods aggregated over the population) using likelihood ratio tests. For Bayesian model comparison, we computed a Laplace approximation to the model evidence (MacKay, 2003) integrating out the free parameters; this analysis requires a prior over the parameters, which we took to be Beta(1.1,1.1) for the learning rates, λ and *w*, Normal(0,1) for *p*, and Gamma(1.2,5) for the softmax temperatures, selected so as to be uninformative over the parameter ranges we have seen in previous studies, and to roll off smoothly at parametric boundaries. We also fit the model of Stephan et al. (2009), which takes model identity as a random effect, by submitting the Laplace-approximated log model evidences to the spm_BMS routine from spm8.

Thus, we performed all behavioral analyses assuming the parameters (and in some cases the model identity) as random effects across subjects. However, to generate regressors for neural analyses on a common scale, we refit the algorithm to the choices taking only *w* as a random effect, instantiated once per subject, and assuming common values for the other parameters. This is because in these sorts of algorithms, noise and variation in parameter estimates from subject to subject results, effectively, in a rescaling of regressors between subjects, which suppresses the significance of neural effects in a subsequent second-level fMRI analysis, producing poor results (Daw, in press; Daw et al., 2006b; Gershman et al., 2009; Schonberg et al., 2007; Schonberg et al., 2010).

### fMRI procedures

Functional imaging was conducted using a 1.5T Siemens Sonata MRI scanner to acquire gradient echo T2*-weighted echo-planar images (EPI) with blood oxygenation level dependent (BOLD) contrast. Standard preprocessing was performed; see Supplemental Experimental Procedures for full details of preprocessing and acquisition.

### fMRI analysis

The fMRI analysis was based around the timeseries of model-free and model-based RPEs as generated from the simulation of the model over each subject’s experiences. We defined two parametric regressors – the model-free RPE, and the difference between the model-free and model-based RPEs. The latter regressor characterizes how net BOLD activity would differ if it were correlated with model-based RPEs or any weighted mixture of both. For each trial, the RPE timeseries were entered as parametric regressors modulating impulse events at the second-stage onset and reward receipt. To test the correspondence between behavioral and neural estimates of the model-based effect, we also included the per-subject estimate of the model-based effect (*w*, above) from the behavioral fits as a second-level covariate for the difference regressor. A full description of the analysis is given in Supplemental Experimental Procedures.

For display purposes, we render activations at an uncorrected threshold of p<.001 (except relaxing this in one case to p<.005), overlaid on the average of subjects’ normalized structural images. For all reported statistics, we subjected these uncorrected maps to cluster-level correction for family-wise error due to multiple comparisons over the whole brain, or, in a few cases (noted specifically) over a small volume defined by an anatomical mask of bilateral nucleus accumbens. This mask was hand-drawn on the subject-averaged structural image, according to the guidelines of Breiter et al. (Ballmaier et al., 2004; Breiter et al., 1997; Schonberg et al., 2010), notably, defining the nucleus’ superior border by a line connecting the most ventral point of the lateral ventricle to the most ventral point of the internal capsule at the level of the putamen. Conjunction inference was by the minimum *t*-statistic (Nichols et al., 2005) using the conjunction null hypothesis. The difference regressor was orthogonalized against the RPE regressor, so that up to minor correlation that can be reintroduced by whitening and filtering, it captured only residual variation in BOLD activity not otherwise explained by the model-free RPE. However, note that conjunction inference via the minimum *t*-statistic is valid even when the conjoined contrasts are not independent (Nichols et al., 2005).

### ROI analyses

We also used the right-hemisphere portion of the mask of nucleus accumbens (right being the side on which we have previously observed stronger RPE activity; e.g. Daw et al., 2006b; Wittmann et al., 2008) to define the region of interest for two analyses conducted with the MarsBaR ROI toolbox (Brett et al., 2002). First, average activity from the region was extracted and subjected to the same analysis as described above, to produce Figure 3f. Second, the activity from the region was subject to a second regression analysis using a different design, which tagged the first-stage onset of each trial with an impulse regressor of one of five types: switches (trials on which the opposite first-stage choice was made than on the previous trial), and, for stays, four types of events modelling all combinations of the factors reward vs. nonreward and common vs. rare transition in the previous trial. An additional nuisance regressor was included at the time of outcomes. Per-subject effect sizes for the four “stay” regressors were subject to a 2×2 repeated-measure ANOVA, and, additionally, the value for each subject of the contrast measuring the interaction of the two factors ([reward/common minus nonreward/common] minus [reward/rare minus nonreward/rare]) was correlated with the weight given to model-based values (the estimated parameter *w*) from the behavioral fit.

## Supplementary Material

01 Supplemental Experimental Procedures

## Figures and Tables

**Table 1:** Best-fitting parameter estimates, shown as median + quartiles across subjects. Also shown are medians and quartiles for the negative log-likelihood (−LL) of the data at the best fitting parameters, and a pseudo-*r*^2^ statistic (p-r^2^), a normalized measure of the degree to which the model explained the choice data.

	β_1_	β_2_	α_1_	α_2_	λ	p	w	−LL	p-r^2^
25^th^ pctile	2.76	2.69	0.46	0.21	0.41	0.02	0.29	167.74	.17
**median**	**5.19**	**3.69**	**0.54**	**0.42**	**0.57**	**0.11**	**0.39**	**200.55**	**.26**
75^th^ pctile	7.45	5.16	0.87	0.71	0.94	0.22	0.59	228.22	.40

**Table 2:** Model comparisons between full (hybrid) model and its special cases. Shown for each model are raw negative log likelihood (−LL); the number of subjects favoring the hybrid model on a likelihood ratio test (p<.05); test statistic and p value for a likelihood ratio test against the hybrid model, aggregated across subjects; the negative log model evidence −log(P(M|D)); the number of subjects favouring the hybrid model according to the model evidence; the log Bayes factor favouring the hybrid model, in the aggregate over subjects; and the Bayesian exceedance probability (Stephan et al. 2009) or probability that each model is the most common among the five over the population.

	classical			Bayesian			
	−LL	# favoring hybrid	agg. LRT favoring hybrid	−log(P(M|D))	# favoring hybrid	agg. log Bayes factor favoring hybrid	exceedance prob
hybrid	3364	-	-	3564	-	-	.92
TD only	3418	5	χ^2^_17_ = 108 p < 5e-15	3594	11	30.0	.031
model-based only	3501	14	χ^2^_51_ = 273 p < 5e-16	3646	15	82.4	.0019
λ=0	3452	14	χ^2^_17_ = 176 p < 5e-16	3627	16	62.9	.0012
λ=1	3392	4	χ^2^_17_ = 54.5 p < 1e-5	3573	8	8.87	.049

**Table 3:** Mixed effects parameter estimates used for fMRI regressors.

β_1_	β_2_	α_1_	α_2_	λ	p	w	−LL	p-r^2^
4.23	2.95	0.70	0.40	0.63	0.17	mean 0.51 SD 0.31	3702	.22

## References

[R1] Adams C (1982). Variations in the sensitivity of
instrumental responding to reinforcer devaluation. The
Quarterly Journal of Experimental Psychology Section
B.

[R2] Balleine B, O’Doherty J (2010). Human and rodent homologies in
action control: corticostriatal determinants of goal-directed and habitual
action. Neuropsychopharmacology.

[R3] Balleine BW, Daw ND, O’Doherty JP, Glimcher PW, Camerer C, Poldrack RA, Fehr E (2008). Multiple forms of value learning
and the function of dopamine. Neuroeconomics: Decision
Making and the Brain.

[R4] Ballmaier M, Toga A, Siddarth P, Blanton R, Levitt J, Lee M, Caplan R (2004). Thought disorder and nucleus
accumbens in childhood: a structural MRI
study. Psychiatry
Res.

[R5] Barto A, Sutton R, Anderson C (1983). Neuronlike adaptive elements that
can solve difficult learning control problems. IEEE
Transactions on systems, man, and
cybernetics.

[R6] Barto AG, Houk JC, Beiser DG (1995). Adaptive Critics and the Basal
Ganglia. Models of Information Processing in the Basal
Ganglia.

[R7] Bates D, Maechler M (2010). lme4: linear mixed effects models
using S4 classes. R package version
0.999375.

[R8] Bayer HM, Glimcher PW (2005). Midbrain dopamine neurons encode
a quantitative reward prediction error
signal. Neuron.

[R9] Berns GS, McClure SM, Pagnoni G, Montague PR (2001). Predictability modulates human
brain response to reward. J
Neurosci.

[R10] Bertin M, Schweighofer N, Doya K (2007). Multiple model-based
reinforcement learning explains dopamine neuronal
activity. Neural
Netw.

[R11] Breiter H, Gollub R, Weisskoff R, Kennedy D, Makris N, Berke J, Goodman J, Kantor H, Gastfriend D, Riorden J (1997). Acute effects of cocaine on human
brain activity and
emotion. Neuron.

[R12] Brett M, Anton J-L, Valabregue R, Poline J-B (2002). Region of interest analysis using
an SPM toolbox.

[R13] Bromberg-Martin E, Matsumoto M, Hong S, Hikosaka O (2010). A pallidus-habenula-dopamine
pathway signals inferred stimulus values. J
Neurophysiol.

[R14] Daw ND, Phelps EA, Robbins TW, Delgado M Trial-by-trial data
analysis using computational models. Affect, Learning
and Decision Making, Attention and
Performance.

[R15] Daw ND, Courville AC, Touretzky DS (2006a). Representation and timing in
theories of the dopamine system. Neural
Comput.

[R16] Daw ND, Niv Y, Dayan P (2005). Uncertainty-based competition
between prefrontal and dorsolateral striatal systems for behavioral
control. Nat
Neurosci.

[R17] Daw ND, O’Doherty JP, Dayan P, Seymour B, Dolan RJ (2006b). Cortical substrates for
exploratory decisions in
humans. Nature.

[R18] Delgado M, Gillis M, Phelps E (2008). Regulating the expectation of
reward via cognitive strategies. Nature
neuroscience.

[R19] Delgado MR, Nystrom LE, Fissell C, Noll DC, Fiez JA (2000). Tracking the hemodynamic
responses to reward and punishment in the striatum. J
Neurophysiol.

[R20] Dickinson A (1985). Actions and habits: the
development of behavioural autonomy. Philosophical
Transactions of the Royal Society of London. Series B, Biological
Sciences.

[R21] Dickinson A, Balleine B, Paschler H, Gallistel R (2002). The role of learning in the
operation of motivational systems. Stevens’
Handbook of Experimental Psychology, Third Edition, Vol.3: Learning,
Motivation, and Emotion.

[R22] Doeller CF, Burgess N (2008). Distinct error-correcting and
incidental learning of location relative to landmarks and
boundaries. Proc Natl Acad Sci U S
A.

[R23] Doeller CF, King JA, Burgess N (2008). Parallel striatal and hippocampal
systems for landmarks and boundaries in spatial
memory. Proc Natl Acad Sci U S
A.

[R24] Doll B, Jacobs W, Sanfey A, Frank M (2009). Instructional control of
reinforcement learning: A behavioral and neurocomputational
investigation. Brain
research.

[R25] Doya K (1999). What are the computations of the
cerebellum, the basal ganglia and the cerebral
cortex?. Neural
Netw.

[R26] Doya K, Samejima K, Katagiri K, Kawato M (2002). Multiple model-based
reinforcement learning. Neural
Comput.

[R27] Everitt B, Robbins T (2005). Neural systems of reinforcement
for drug addiction: from actions to habits to
compulsion. Nat
Neurosci.

[R28] Fitzgerald T, Seymour B, Bach D, Dolan R (2010). Differential neural substrates
for learnt and described value and risk. Current
Biology.

[R29] Foster DJ, Wilson MA (2006). Reverse replay of behavioural
sequences in hippocampal place cells during the awake
state. Nature.

[R30] Frank M, Moustafa A, Haughey H, Curran T, Hutchison K (2007). Genetic triple dissociation
reveals multiple roles for dopamine in reinforcement
learning. Proceedings of the National Academy of
Sciences.

[R31] Friston K, Josephs O, Rees G, Turner R (1998). Nonlinear event-related responses
in fMRI. Magn Reson
Med.

[R32] Friston K, Worsley K, Frackowiak R, Mazziotta J, Evans A (1993). Assessing the significance of
focal activations using their spatial extent. Human
brain
mapping.

[R33] Fu W, Anderson J (2008). Solving the credit assignment
problem: Explicit and implicit learning of action sequences with
probabilistic outcomes. Psychological
Research.

[R34] Gershman S, Pesaran B, Daw N (2009). Human reinforcement learning
subdivides structured action spaces by learning effector-specific
values. J
Neurosci.

[R35] Gläscher J, Daw N, Dayan P, O’Doherty J (2010). States versus rewards:
dissociable neural prediction error signals underlying model-based and
model-free reinforcement
learning. Neuron.

[R36] Hampton AN, Bossaerts P, O’Doherty JP (2006). The role of the ventromedial
prefrontal cortex in abstract state-based inference during decision making
in humans. J
Neurosci.

[R37] Hampton AN, Bossaerts P, O’Doherty JP (2008). Neural correlates of
mentalizing-related computations during strategic interactions in
humans. Proc Natl Acad Sci U S
A.

[R38] Hare TA, O’Doherty J, Camerer CF, Schultz W, Rangel A (2008). Dissociating the role of the
orbitofrontal cortex and the striatum in the computation of goal values and
prediction errors. J
Neurosci.

[R39] Hassabis D, Maguire E (2007). Deconstructing episodic memory
with construction. Trends in Cognitive
Sciences.

[R40] Holmes A, Friston K (1998). Generalisability, random effects
& population
inference. Neuroimage.

[R41] Ito M, Doya K (2009). Validation of decision-making
models and analysis of decision variables in the rat basal
ganglia. J
Neurosci.

[R42] Johnson A, Redish A (2005). Hippocampal replay contributes to
within session learning in a temporal difference reinforcement learning
model. Neural
Networks.

[R43] Johnson A, Redish AD (2007). Neural ensembles in CA3
transiently encode paths forward of the animal at a decision
point. J
Neurosci.

[R44] Kable J, Glimcher P (2007). The neural correlates of
subjective value during intertemporal choice. Nat
Neurosci.

[R45] Kahneman D (2003). A perspective on judgment and
choice: Mapping bounded rationality. American
psychologist.

[R46] Killcross S, Coutureau E (2003). Coordination of actions and
habits in the medial prefrontal cortex of rats. Cereb
Cortex.

[R47] Kim H, Sul JH, Huh N, Lee D, Jung MW (2009). Role of striatum in updating
values of chosen actions. J
Neurosci.

[R48] Knutson B, Gibbs SE (2007). Linking nucleus accumbens
dopamine and blood oxygenation. Psychopharmacology
(Berl).

[R49] Knutson B, Rick S, Wimmer GE, Prelec D, Loewenstein G (2007). Neural predictors of
purchases. Neuron.

[R50] Knutson B, Westdorp A, Kaiser E, Hommer D (2000). FMRI visualization of brain
activity during a monetary incentive delay
task. Neuroimage.

[R51] Lau B, Glimcher PW (2005). Dynamic response-by-response
models of matching behavior in rhesus monkeys. J Exp
Anal
Behav.

[R52] Loewenstein G, O’Donoghue T (2004). Animal spirits: Affective and
deliberative processes in economic behavior. Working
Paper 04–14.

[R53] Lohrenz T, McCabe K, Camerer CF, Montague PR (2007). Neural signature of fictive
learning signals in a sequential investment task. Proc
Natl Acad Sci U S
A.

[R54] MacKay DJC (2003). Information theory, inference, and
learning algorithms.

[R55] Maia T (2010). Two-factor theory, the
actor-critic model, and conditioned avoidance. Learn
Behav.

[R56] McClure SM, Berns GS, Montague PR (2003a). Temporal prediction errors in a
passive learning task activate human
striatum. Neuron.

[R57] McClure SM, Daw ND, Montague PR (2003b). A computational substrate for
incentive salience. Trends
Neurosci.

[R58] Montague PR, Dayan P, Person C, Sejnowski TJ (1995). Bee foraging in uncertain
environments using predictive hebbian
learning. Nature.

[R59] Montague PR, Dayan P, Sejnowski TJ (1996). A framework for mesencephalic
dopamine systems based on predictive Hebbian
learning. J
Neurosci.

[R60] Moore A, Atkeson C (1993). Prioritized sweeping:
Reinforcement learning with less data and less
time. Machine
Learning.

[R61] Morris G, Nevet A, Arkadir D, Vaadia E, Bergman H (2006). Midbrain dopamine neurons encode
decisions for future action. Nat
Neurosci.

[R62] Nichols T, Brett M, Andersson J, Wager T, Poline J (2005). Valid conjunction inference with
the minimum
statistic. Neuroimage.

[R63] Niv Y, Joel D, Dayan P (2006). A normative perspective on
motivation. Trends Cogn
Sci.

[R64] O’Doherty JP (2004). Reward representations and
reward-related learning in the human brain: insights from
neuroimaging. Curr Opin
Neurobiol.

[R65] O’Doherty JP, Dayan P, Friston K, Critchley H, Dolan RJ (2003). Temporal difference models and
reward-related learning in the human
brain. Neuron.

[R66] O’Doherty JP, Hampton A, Kim H (2007). Model-based fMRI and its
application to reward learning and decision
making. Ann N Y Acad
Sci.

[R67] Pessiglione M, Seymour B, Flandin G, Dolan RJ, Frith CD (2006). Dopamine-dependent prediction
errors underpin reward-seeking behaviour in
humans. Nature.

[R68] Peters J, Buchel C (2009). Overlapping and distinct neural
systems code for subjective value during intertemporal and risky decision
making. J
Neurosci.

[R69] Plassmann H, O’Doherty J, Rangel A (2007). Orbitofrontal cortex encodes
willingness to pay in everyday economic
transactions. J
Neurosci.

[R70] Poldrack RA, Clark J, Pare-Blagoev EJ, Shohamy D, Creso Moyano J, Myers C, Gluck MA (2001). Interactive memory systems in the
human
brain. Nature.

[R71] Preuschoff K, Bossaerts P, Quartz SR (2006). Neural differentiation of
expected reward and risk in human subcortical
structures. Neuron.

[R72] Rangel A, Camerer C, Montague PR (2008). A framework for studying the
neurobiology of value-based decision making. Nat Rev
Neurosci.

[R73] Redish A, Jensen S, Johnson A (2008). A unified framework for
addiction: vulnerabilities in the decision
process. Behav Brain
Sci.

[R74] Rummery G, Niranjan M (1994). On-line Q-learning using
connectionist systems. Technical Report
CUED/F-INFENG/TR 166, Engineering Department, Cambridge
University.

[R75] Schacter D, Addis D, Buckner R (2007). Remembering the past to imagine
the future: the prospective brain. Nat Rev
Neurosci.

[R76] Schonberg T, Daw ND, Joel D, O’Doherty JP (2007). Reinforcement learning signals in
the human striatum distinguish learners from nonlearners during reward-based
decision making. J
Neurosci.

[R77] Schonberg T, O’Doherty J, Joel D, Inzelberg R, Segev Y, Daw N (2010). Selective impairment of
prediction error signaling in human dorsolateral but not ventral striatum in
Parkinson’s disease patients: evidence from a model-based fMRI
study. Neuroimage.

[R78] Schultz W, Dayan P, Montague PR (1997). A neural substrate of prediction
and
reward. Science.

[R79] Seymour B, O’Doherty JP, Dayan P, Koltzenburg M, Jones AK, Dolan RJ, Friston KJ, Frackowiak RS (2004). Temporal difference models
describe higher-order learning in
humans. Nature.

[R80] Sloman S (1996). The empirical case for two
systems of reasoning. Psychological
bulletin.

[R81] Stephan KE, Penny WD, Daunizeau J, Moran RJ, Friston KJ (2009). Bayesian model selection for
group
studies. Neuroimage.

[R82] Suri R, Schultz W (1999). A neural network model with
dopamine-like reinforcement signal that learns a spatial delayed response
task. Neuroscience.

[R83] Sutton R (1990). Integrated architectures for learning,
planning, and reacting based on approximating dynamic
programming.

[R84] Tanaka SC, Doya K, Okada G, Ueda K, Okamoto Y, Yamawaki S (2004). Prediction of immediate and
future rewards differentially recruits cortico-basal ganglia
loops. Nat
Neurosci.

[R85] Thorndike EL (1911). Animal intelligence; experimental
studies.

[R86] Tolman E (1948). Cognitive maps in rats and
men. Psychological
Review.

[R87] Tom SM, Fox CR, Trepel C, Poldrack RA (2007). The neural basis of loss aversion
in decision-making under
risk. Science.

[R88] Valentin VV, Dickinson A, O’Doherty JP (2007). Determining the neural substrates
of goal-directed learning in the human brain. J
Neurosci.

[R89] Venkatraman V, Payne JW, Bettman JR, Luce MF, Huettel SA (2009). Separate neural mechanisms
underlie choices and strategic preferences in risky decision
making. Neuron.

[R90] Wittmann BC, Daw ND, Seymour B, Dolan RJ (2008). Striatal activity underlies
novelty-based choice in
humans. Neuron.

[R91] Yin HH, Knowlton BJ, Balleine BW (2004). Lesions of dorsolateral striatum
preserve outcome expectancy but disrupt habit formation in instrumental
learning. Eur J
Neurosci.

[R92] Yin HH, Ostlund SB, Knowlton BJ, Balleine BW (2005). The role of the dorsomedial
striatum in instrumental conditioning. Eur J
Neurosci.

