# Supplementary material for: Model-based influences on humans’ choices and striatal prediction errors
Source: Neuron. Author manuscript; Available in PMC 2012 Mar 24. (PMC3077926; doi:10.1016/j.neuron.2011.02.027)
Supplement: 01 Supplemental Experimental Procedures [file NIHMS280176-supplement-01.pdf]

Supplemental material for “Model-based influences on humans’ choices and striatal prediction errors”

Nathaniel D. Daw, Samuel J. Gershman, Ben Seymour, Peter Dayan & Raymond J. Dolan

Supplemental experimental procedures

Computational model of behavior

The task consists of three states (first stage: s_A; second stage: s_B and s_C), each with two actions (a_A and a_B). The goal of both the model-based and model-free subcomponents of the algorithm is to learn a state-action value function $Q(s,a)$ mapping each state-action pair to its expected future value. On trial *t*, we denote the first-stage state (always s_A) by $s_{1,t}$, the second-stage state by $s_{2,t}$, the first- and second-stage actions by $a_{1,t}$ and $a_{2,t}$, and the first- and second-stage rewards as $r_{1,t}$ (always zero) and $r_{2,t}$.

The model free algorithm was SARSA(λ) temporal difference learning (Rummery and Niranjan, 1994). At each stage *i* of each trial *t*, the value for the visited state-action pair was updated according to:

$$Q_{TD}(s_{i,t}, a_{i,t}) = Q_{TD}(s_{i,t}, a_{i,t}) + \alpha_i \delta_{i,t}$$

where

$$\delta_{i,t} = r_{i,t} + Q_{TD}(s_{i+1,t}, a_{i+1,t}) - Q_{TD}(s_{i,t}, a_{i,t}) \quad [1]$$

and α_i are free learning-rate parameters. (We allow different learning rates α_1 and α_2 for the two task stages, to ensure our primary analyses of top-level effects are not affected by any potential difference in learning or behavior between the stages. Such effects might arise if there were differences in learning from state transitions vs rewards, and because any second-level state/action is sampled less frequently than the top-level ones.) Note that, for the first-stage choice, $r_{1,t} = 0$ and the RPE is instead driven by the second-stage value, $Q_{TD}(s_{2,t}, a_{2,t})$; conversely at the second stage, we define $Q_{TD}(s_{3,t}, a_{3,t}) = 0$, since there is no further value in the trial apart from the immediate reward $r_{2,t}$. Since this task has only two stages per trial, the only effect of the eligibility parameter λ (Sutton and Barto, 1998) is, at the end of each trial, to modulate an additional stage-skipping update of the first-stage action by the second-stage RPE, $Q_{TD}(s_{1,t}, a_{1,t}) = Q_{TD}(s_{1,t}, a_{1,t}) + \alpha_1 \lambda \delta_{2,t}$. Note that this model assumes that eligibility traces are cleared between episodes (i.e., that eligibility does not carry over from trial to trial), which appears to be a reasonable simplification since eligibility carryover would be inconsistent with the episodic structure of the task, about which subjects were instructed; though see Walton et al. 2010)

In general, a model-based RL algorithm works by learning a transition function (mapping state-action pairs to a probability distribution over the subsequent state), and immediate reward values for each state, then computing cumulative state-action values by iterative expectation over these. Specialized to

the structure of the current task, this amounts to, first, simply deciding which first-stage action maps to which second-stage state (since subjects were instructed that this was the structure of the transition contingencies), and second, learning immediate reward values for each of the second-stage actions (the immediate rewards at the first stage being always zero).

We characterized transition learning by assuming subjects simply chose between the two possibilities: $P(s_B|s_A, a_A) = 0.7, P(s_C|s_A, a_B) = 0.7$, or, vice versa $P(s_B|s_A, a_A) = 0.3, P(s_C|s_A, a_B) = 0.3$ (with $P(s_C|s_A, a_A) = 1 - P(s_B|s_A, a_A)$ and $P(s_B|s_B, a_A) = 1 - P(s_C|s_A, a_B)$, according to whether more transitions had so far occurred to s_B following a_A plus s_C following a_B, or, vice versa, to s_C following a_A plus s_B following a_B. (In analyses not reported here, we verified that this scheme, which settles on the true transition matrix after the first few trials and is consistent with subjects' instructions, fit their choices better than traditional incremental learning schemes for estimating transition matrices. The specific values 0.7/0.3 are chosen without loss of generality; if these are changed, other free parameters of the algorithm will rescale to give the same overall choice likelihood.)

At the second-stage (the only one where immediate rewards were offered), the problem of learning immediate rewards is equivalent to that for TD above, since $Q_{TD}(s_{2,t}, a_{2,t})$ is just an estimate of the immediate reward $r_{2,t}$; with no further stages to anticipate, the SARSA learning rule reduces to a delta-rule for predicting the immediate reward. Thus the two approaches coincide at the second stage, and we define $Q_{MB} = Q_{TD}$ at those states.

Next, using Bellman's equation, we define the model-based values of the first level actions as

$$Q_{MB}(s_A, a_j) = P(s_B|s_A, a_j) \max_{a\in\{a_A,a_B\}} Q_{TD}(s_B, a) + P(s_C|s_A, a_j) \max_{a\in\{a_A,a_B\}} Q_{TD}(s_C, a)$$

and assume these are recomputed at each trial from the current estimates of the transition probabilities and rewards.

Finally, to connect the values to choices, we define net action values at the first stage as the weighted sum of model-based and model-free values $Q_{net}(s_A, a_j) = wQ_{MB}(s_A, a_j) + (1 - w)Q_{TD}(s_A, a_j)$ where *w* is a weighting parameter. At the second stage, $Q_{net} = Q_{MB} = Q_{TD}$. We then assume the probability of a choice is softmax in Q_{net}:

$$P(a_{i,t} = a|s_{i,t}) = \frac{\exp(\beta_i[Q_{net}(s_{i,t}, a) + p \cdot \mathrm{rep}(a)])}{\sum_{a'} \exp(\beta_i[Q_{net}(s_{i,t}, a') + p \cdot \mathrm{rep}(a')])} \quad [2]$$

Here, the free inverse temperature parameters β_i control how deterministic are the choices, and we allow β_1 and β_2 to differ between the stages. (This captures any differences in choice reliability between the stages; note that this also renders redundant a time-discount parameter.) The indicator function $\mathrm{rep}(a)$ is defined as 1 if *a* is a top-stage action and is the same one as was chosen on the previous trial, zero otherwise. Together with the free parameter *p*, this captures first-order perseveration ($p > 0$) or switching ($p < 0$) in the first-stage choices (Lau and Glimcher, 2005; also visible in Figure 2c). We do not include such autocorrelation for the second-stage choices, simply because (since different second-level

states are visited from trial to trial) choice repetition at the second stage is less likely to play a large role, and it is also less clear how best to define it.

In total, the algorithm contains 7 free parameters (β_1, β_2, α_1, α_2, λ, p, w), and nests pure model-based (w = 1, with arbitrary α_1 and λ) and model-free (w = 0) learning as special cases.

For neural analysis, we defined a generalized version of Equation 1, measuring RPEs with respect to net model-based/model-free values Q_{net}:

$$\delta_{net,i,t} = r_{i,t} + Q_{net}(s_{i+1,t}, a_{i+1,t}) - Q_{net}(s_{i,t}, a_{i,t}) \quad [3]$$

and took its partial derivative with respect to the parameter w mixing Q_{TD} and Q_{MB} into Q_{net}, which we refer to as the "difference regresssor" since it is the difference between δ_{net} computed for w=1 and w=0.

fMRI procedures

Functional imaging was conducted using a 1.5T Siemens Sonata MRI scanner to acquire gradient echo T2*-weighted echo-planar images (EPI) with blood oxygenation level dependent (BOLD) contrast. We employed a special pulse sequence designed to optimize functional sensitivity in OFC (Deichmann et al., 2003). This consisted of tilted acquisition in an oblique orientation at 30 degrees to the AC-PC line, as well as application of a preparation pulse with a duration of 1ms and amplitude of -2mT/m in the selection direction. The sequence enabled 36 axial slices of 3mm thickness and 3mm in-plane resolution to be acquired with a repetition time (TR) of 3.24s. Coverage was obtained from the base of the orbitofrontal cortex and medial temporal lobes to the superior border of the dorsal anterior cingulate cortex. Participants were placed in a light head restraint within the scanner to limit head movement during acquisition. A field map was also recorded for distortion correction of the acquired EPI images, using a double echo FLASH sequence (64 oblique transverse slices, slice thickness = 2 mm, gap between slices = 1 mm, TR = 1170 ms, α = 90°, short TE = 10 ms, long TE = 14.76 ms, BW = 260 Hz/pixel, PE direction anterior–posterior, FOV = 192×192 mm^2, matrix size 64 × 64, flow compensation). A T1-weighted structural image was also acquired for each subject.

Preprocessing and data analysis were performed using Statistical Parametric Mapping software implemented in Matlab (SPM5; Wellcome Department of Imaging Neuroscience, Institute of Neurology, London, UK; and SPM8 for final results visualization and multiple comparison correction). Using the FieldMap toolbox (Hutton et al., 2002; Hutton et al., 2004), field maps were estimated from the phase difference between the images acquired at the short and long TEs in the FLASH sequence. The EPI images were corrected for subject motion by realigning them to the first volume, and simultaneously corrected for geometric distortion based on the field map and the interaction of distortion with motion (SPM5 "realign and unwarp"; Andersson et al., 2001; Hutton et al., 2002; Hutton et al., 2004). EPI images were then spatially normalized to the Montreal Neurological Institute template by warping the subject's anatomical image to an SPM segmentation template (SPM5 "segment and normalize") and applying these parameters to the functional images, resampled into 2x2x2 mm sized voxels, and smoothed using an 8 mm Gaussian kernel.

For statistical analysis, the data were scaled voxel-by-voxel onto their global mean and high-pass-filtered using a filter width of 128 secs.

fMRI analysis

The fMRI analysis was based around the timeseries of RPEs as generated from the simulation of the model over each subject's experiences. Note that as defined by Equation 1 above, this error is nonzero at two timepoints: the onset of the second stage, when $\delta_{1,t}$ is realized, and at the reward receipt, when $\delta_{2,t}$ is realized. These two RPEs ostensibly train the values that drive the two choices in the task. In general, RPE can also be defined at a third point, the start of the trial (the onset of stage 1; see Daw et al., 2006; Schonberg et al., 2007; Schonberg et al., 2010), but this is more difficult to define accurately enough to analyze parametrically since it depends on the value expectation prior to trial onset, a quantity which is not assessed behaviorally. Therefore, we do not include this timepoint in our parametric analysis of RPE effects (instead defining nuisance regressors to control variance there), but we separately subject activity at this timepoint to a complementary, factorial analysis as a relatively independent test of our conclusions (see ROI analyses in main text Experimental Procedures).

We included the RPE as a parametric regressor modulating impulse events at the second-stage onset and reward receipt. The regressor, from Equation 1, corresponds to the generalized model-based/model-free RPE (Equation 3) computed for the mixing parameter $w = 0$. We included an additional parametric regressor, defined at the same timepoints, containing the partial derivative of this timeseries with respect to w. Intuitively, the partial derivative captures how the RPE would change if it were computed according to a different value of w (Friston et al., 1998); in this case, it is just the difference between the RPEs computed with respect to model-based and model-free action values. Since this difference is zero at outcome time, but nonzero at the second-stage onset, to exclude the possibility that the difference effect would be confounded by a simple difference in average striatal activity between these two events, we mean-corrected the difference regressor's values at the choicepoint to zero mean within-subject, and also included an additional nuisance onset at the time of outcome reveal so as to capture any difference in mean activity between the choice and outcome events. We included another nuisance onset at thefirst-stage trial onset, modulated by two additional parametric regressors, also treated as nuisance effects: $P\left(a_{1,t}|s_A\right)$ (from Equation 2), as a normalized measure of the first-stage action value (Daw et al., 2006), and its partial derivative with respect to w.

These regressors were then convolved with the canonical hemodynamic response function, and entered into a regression analysis against each subject's fMRI data using SPM. The 6 scan-to-scan motion parameters produced during realignment were included as additional nuisance regressors in the SPM analysis to account for residual effects of scan to scan motion. To enable inference at the group level, the coefficient estimates for the RPE and difference regressors from each individual subject were taken to the second-level to allow random effects group statistics to be computed. To test the correspondence between behavioral and neural estimates of the model-based effect, we also included the per-subject estimate of the model-based effect (w, above) from the behavioral fits as a second-level covariate for the difference regressor.

References

Andersson, J., Hutton, C., Ashburner, J., Turner, R., and Friston, K. (2001). Modeling geometric deformations in EPI time series. Neuroimage *13*, 903-919.

Daw, N.D., O'Doherty, J.P., Dayan, P., Seymour, B., and Dolan, R.J. (2006). Cortical substrates for exploratory decisions in humans. Nature *441*, 876-879.

Deichmann, R., Gottfried, J., Hutton, C., and Turner, R. (2003). Optimized EPI for fMRI studies of the orbitofrontal cortex. Neuroimage *19*, 430-441.

Friston, K., Josephs, O., Rees, G., and Turner, R. (1998). Nonlinear event-related responses in fMRI. Magn Reson Med *39*, 41-52.

Hutton, C., Bork, A., Josephs, O., Deichmann, R., Ashburner, J., and Turner, R. (2002). Image distortion correction in fMRI: A quantitative evaluation. Neuroimage *16*, 217-240.

Hutton, C., Deichmann, R., Turner, R., and Andersson, J.L.R. (2004). Combined correction for geometric distortion and its interaction with head motion in fMRI. In ISMRM 12 (Kyoto, Japan)).

Lau, B., and Glimcher, P.W. (2005). Dynamic response-by-response models of matching behavior in rhesus monkeys. J Exp Anal Behav *84*, 555-579.

Rummery, G., and Niranjan, M. (1994). On-line Q-learning using connectionist systems.

Schonberg, T., Daw, N.D., Joel, D., and O'Doherty, J.P. (2007). Reinforcement learning signals in the human striatum distinguish learners from nonlearners during reward-based decision making. J Neurosci *27*, 12860-12867.

Schonberg, T., O'Doherty, J., Joel, D., Inzelberg, R., Segev, Y., and Daw, N. (2010). Selective impairment of prediction error signaling in human dorsolateral but not ventral striatum in Parkinson's disease patients: evidence from a model-based fMRI study. Neuroimage *49*, 772-781.

Sutton, R.S., and Barto, A.G. (1998). Reinforcement Learning: An Introduction (MIT Press).
